# Prototype Development of an Expert System of Computerized Clinical Guidelines for COVID-19 Diagnosis and Management in Saudi Arabia

**DOI:** 10.3390/ijerph17218066

**Published:** 2020-11-02

**Authors:** Haneen Reda Banjar, Heba Alkhatabi, Nofe Alganmi, Ghaidaa Ibraheem Almouhana

**Affiliations:** 1 Computer Science Department, Faculty of Computing and Information Technology, King Abdulaziz University, Jeddah 80200, Saudi Arabia; nalghanimi@kau.edu.sa (N.A.); galmouhana@stu.kau.edu.sa (G.I.A.); 2 Department of Medical Laboratory Technology, Faculty of Applied Medical Science, King Abdulaziz University, Jeddah 80200, Saudi Arabia; halkhattabi@kau.edu.sa; 3 Center of Excellence in Genomic Medicine Research (CEGMR), King Abdulaziz University, Jeddah 80200, Saudi Arabia

**Keywords:** expert system, clinical guideline, COVID-19 management, mobile track and evolution of expert system

## Abstract

The increasing number of COVID-19 patients has increased health care professionals’ workloads, making the management of dynamic patient information in a timely and comprehensive manner difficult and sometimes impossible. Compounding this problem is a lack of health care professionals and trained medical staff to handle the increased number of patients. Although Saudi Arabia has recently improved the quality of its health services, there is still no suitable intelligent system that can help health practitioners follow the clinical guidelines and automated risk assessment and treatment plan remotely, which would allow for the effective follow-up of patients of COVID-19. The proposed system includes five sub-systems: an information management system, a knowledge-based expert system, adaptive learning, a notification and follow-up system, and a mobile tracker system. This study shows that, to control epidemics, there is a method to overcome the shortage of specialists in the management of infections in Saudi Arabia, both today and in the future. The availability of computerized clinical guidance and an up-to-date knowledge base play a role in Saudi health organizations, which may not have to constantly train their physician staff and may no longer have to rely on international experts, since the expert system can offer clinicians all the information necessary to treat their patients.

## 1. Introduction

COVID-19 is a new human infectious disease caused by Severe Acute Respiratory Syndrome Coronavirus 2 (SARS-CoV-2), which characteristically manifests as respiratory symptoms and is transmissible from human to human. The possible spread of COVID-19 worldwide has been described by the World Health Organization (WHO, Geneva, Switzerland) as an international public health emergency [1]. This disaster hit the globe at the end of 2019 and has affected more than 200 countries. The disease has resulted in about 5,939,234 confirmed cases to date. As of May 2020, about 367,255 individuals have died [2]. With rising numbers of patients with COVID-19, the world is faced with serious problems in providing high-quality health care to people at a reasonable cost. Therefore, patients are increasingly burdening the work of health care professionals, who are often unable to handle dynamic patient information in a timely manner and in a comprehensive way.

Clinical guidelines are standardized statements designed to assist physicians and patients in deciding on appropriate treatment in certain situations [3]. A set of computerized clinical guidelines is one of the ways in which the clinical and scientific community tries to address information excess, reduce failures, and quickly transpose research findings into clinical care. There is a need for computerized clinical guidelines to promote the scientific homogeneity and consistency of health decisions and interventions in daily clinical practice.

In the 1980s, the dominant intelligent technology consisted of expert systems that aimed to simulate human expertise and provide solutions based on current knowledge [4]. Subsequently, the use of expert systems has expanded in the medical field for the diagnosis, management, and treatment of diseases [5]. However, handling the vast amount of various medical information and the continuous updating of medical records in daily clinical practice might result in mistakes. This inefficiency can be addressed by the evolution of expert systems [6] that monitor new patient data and automatically suggest proper clinical directives to be applied or provide clinician assistance and recommendations without the support of a knowledge engineer.

Several studies have been conducted using artificial intelligence techniques to assist health care workers during the COVID-19 crisis [7]. One is an interesting study developed and tested on nomograms and machine learning models for COVID-19 patients during hospital admissions for severity risk assessment and triage [8]. The model was validated by six cohorts and achieved accuracies ranging from 81% to 97%. The clinical parameters used in this study can be validated on other datasets from Saudi Arabia, and their method can be integrated into our proposed system. Another study by Liu et al. [9] developed a COVID-19 risk assessment decision support system for general practitioners. The system was a well-designed mobile information system that was used extensively by Chinese GPs in Zhejiang Province during the COVID-19 outbreak. However, they used Chinese interfaces, which are not applicable in Saudi Arabia, and the automated procedures of the guidelines were followed only by the GPs. An expert system for COVID-19 diagnosis using symptoms was implemented and assessed by one doctor [10]. In this expert system, she monitored the symptoms of patients who had not been admitted to hospital. Thus, since a variety of cases, such as suspected cases and confirmed cases, may determine admission to a general hospital or isolation in a designated hospital, there is need for a complete intelligent system for COVID-19 diagnosis and management. During the COVID-19 epidemic, these models and systems have taken into account major problems from a range of perspectives and have been used and published over a short time, which has been important in the fight against the epidemic.

Saudi Arabia Vision 2030 notes that the ability of medical facilities and hospitals should be increased and properly implemented and that the quality of health services, both therapeutic and preventive, should be improved [11]. Indeed, four Saudi systems have recently been introduced to support COVID-19 diagnosis and management: First, the Health Electronics Surveillance Network (HESN) is a web-based e-health solution that is an integral, flexible system able to accommodate all Saudi public health programs and the Public Health Information System (PHIS), which is designed to better support public health professionals throughout the Kingdom [12]. Second, Rest Assured (Tataman) [13] is a Saudi Arabian Ministry of Health (MOH) application for protecting and providing health care to residents referred to domestic or quarantine isolation for ensuring their safety and improving recuperation processes. Tataman provides services to support arrivals from abroad, suspected cases, contacts with confirmed cases, and confirmed cases in domestic isolation or quarantine. The services include a library for education, the results of swabs tests, updated contact information of confirmed cases, daily health statuses, links to support for epidemiological research, a contact service center and request assistance, a health isolation countdown indicator, and SMS notifications. Third, Tabaud is one of the technological solutions for monitoring the spread of the coronavirus. The application lets users know whether they have contacted people who have been reported to have coronavirus infection or not. In addition, if confirmed cases are found via the app, it sends users alerts. Finally, Tawakkalna was created to display its users’ health status with the highest degree of safety and privacy by color-coding. The app also enables individuals to contribute to breaking the infection chain by identifying contaminated cases or meetings that breach measures of precaution. All three applications are used to control COVID-19 in Saudi Arabia, and the HESN website is currently used to collect information about confirmed COVID-19 cases.

The proposed system in our research can be an extension of the current apps and systems designed to enhance health care and unify actions taken for the diagnosis and management of COVID-19 patients after staying in private or public Saudi hospitals. The use of WHO clinical guidelines could facilitate access to existing standards and medical vocabularies that enable semantic and technical interoperability between hospitals.

However, to the best of our knowledge, there is still no suitable intelligent system in Saudi Arabia that can help health practitioners follow the clinical guidelines and automated risk assessment and treatment plan to manage the patients and follow-up patients of COVID-19 effectively at hospitals. There is currently no option to adopt sets of new clinical rules that can be discovered automatically from stored data or to locate mobile numbers to warn healthy individuals to avoid places that have been visited by suspected cases.

In this paper, we propose a prototype for COVID-19 diagnosis and management in Saudi Arabia. This design can monitor COVID-19 patients and reduce the burden on medical practitioners and the costs of health care. Clinical guidelines can be used as expert knowledge to build up an expert system. The proposed prototype can also evolve with dynamic knowledge by learning new knowledge adaptively from the integration of new clinical guidelines, with an electronic health record (EHR) and a health organizational workflow, without the aid of a knowledge engineer. In addition, risk classification scores can be integrated to distinguish patients according to the severity of risk and can provide patient triage at hospital admission (e.g., home or mobile hospital isolation, hospitalization, or intensive care unit (ICU)). Messaging notifications and targeted content that trigger action at times of importance can be automated.

The following key issues need to be resolved: What relevant domain knowledge is essential for diagnosing and managing COVID-19? What are the appropriate technologies to develop an intelligent system with the ability to evolve diagnostic and management guidelines for COVID-19?

### COVID-19 Diagnosis and Management in Saudi Arabia

According to the Saudi MOH [14], suspected COVID-19 cases are either confirmed cases (asymptomatic and symptomatic) or negative cases (see Supplementary Materials S1 for a COVID-19 case definition). Diagnosis is based on the combination of epidemiological parameters (contact during incubation), the presence of clinical symptoms, and laboratory (nucleic acid amplification tests) and chest imaging tests based on clinical imaging (see Supplementary Materials S1 for COVID-19 diagnosis). The clinical classification is mild, moderate, severe, and critical cases, and pregnant, elder, children, and neonate (see Supplementary Materials S2 for clinical classification of confirmed cases). About 80% of COVID-19 patients have a mild disease, and symptoms normally disappear within two weeks, while the remaining cases can continue and require hospitalization and increased medical assistance [8]. Therapy recommendations vary among countries [15]. The WHO recommendations are generic and consist of symptom management and promoting treatment for pediatric, pregnant, and underlying morbidity patients (see Supplementary Materials S2 for COVID-19 management). No approved treatment for COVID-19 is available; it is only recommended that each patient’s needs are provided [15]. However, the country-wide treatment protocols are similar, including Lopinavir/Ritonavir, Ribavirin, interferon beta-1b, Remdesivir, favipiravir, and tocilizumab [16]. The WHO recommendations also suggest that empiric antimicrobial therapy should be conducted for severe cases, with mechanical ventilation carried out according to the clinical condition of the patient. Decisions are based on the risk classification for the confirmed cases using the Confusion, Urea nitrogen, Respiratory rate, Blood pressure, and 65 years of age and older (CURB-65) score [17]. 

## 2. Materials and Methods

### 2.1. Architecture Overview

The software development life cycle (SDLC) is a framework that defines the stages involved in the development of software at each level. The proposed intelligent system for COVID-19 diagnosis and management includes five cases of users: health care workers, health care practitioners, patients, lab managers or radiologists, and healthy persons. The framework includes five sub-systems: an information management system, an expert system, adaptive learning, a notification and follow-up system, and a mobile tracker system.

**Figure 1:**
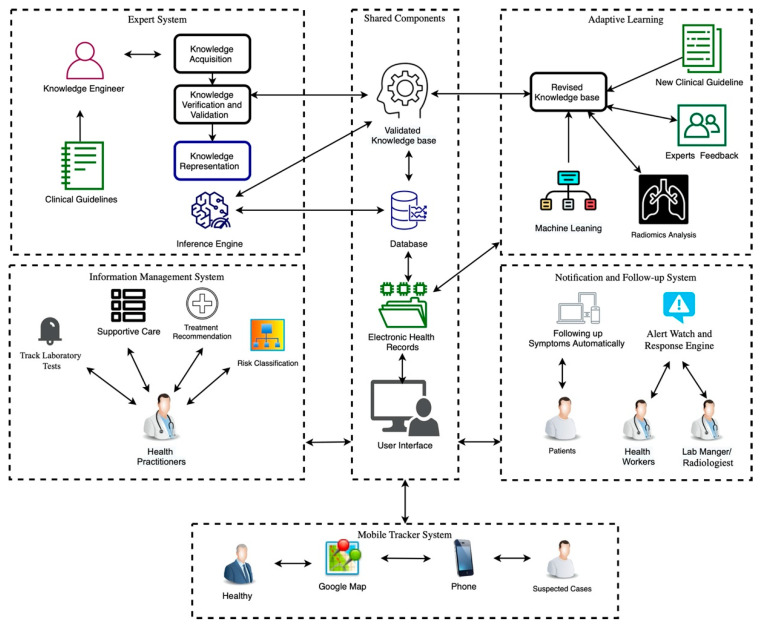
The system architecture. The framework includes five sub-systems. (i) The information management system includes laboratory tests, supportive care, treatment recommendations, and risk classifications. The health practitioners can request information from the database and the expert system. (ii) The expert system includes knowledge acquisition, knowledge verification and validation, and knowledge representation after the information is collected by a knowledge engineer. The knowledge can be collected from two resources: clinical guidelines and the adaptive learning system. (iii) Adaptive learning includes the revised knowledge base. This new knowledge can be collected from new clinical guidelines, radiomics analysis of chest images, and rules discovered by decision trees (CART) from the electronic health records of previous patients. This new knowledge will be verified by experts, and his/her feedback and permission will add it to the validated knowledge base after the knowledge is revised. (iv) The notification and follow-up system includes the alert watch and the response engine software to alert health workers and lab managers or radiologists about abnormal tests, while patients can also follow up with his/her symptoms automatically. (v) The mobile tracker system includes Google Maps and a phone number list of suspected cases. This system will display a Google map showing places that can be visited by healthy persons—i.e., where there have been suspected cases.

The system architecture is shown in Figure 1. The main step is the use of guidelines published by the Saudi MOH [16]. The COVID-19 management procedures are used to build the information management system with the expert system and to map the possible recommendations based on the patient information in the EHR. Adaptive learning evolves new knowledge through the integration of new clinical guidelines with the EHR and health organizational workflow, without the aid of a knowledge engineer. The expert system recommendations include risk classification, a treatment plan, supportive care, recommended laboratory tests, and chest images. A notification and follow-up system tracks the EHR and produces suitable notifications by recommending that the symptoms be described daily, repeats the test, alerts health workers of any new behavior in symptoms or changes in asymptomatic cases, and notifies health practitioners of abnormal test results through EHR-based alerts. Finally, a mobile tracker is integrated into the system to monitor suspected cases by locating their phone number. The system enables healthy individuals to check whether suspected cases are associated with specific sites. This helps in isolating suspected cases from healthy cases. In the following, each subproject and its development phases are described.

### 2.2. Information Management System

An information management system (IMS) is an organizational network of people, behaviors, and devices that provides managers with the information they need [18]. This concept can be applied in the health sector as the main system for retrieving queries, displaying reports and lists, and storing patients’ data and their health services. The database stores the patient’s information; clinical information; hospitalization information; epidemiological information; contact exposure; animal exposure; a list of the patient’s contacts; personal details; and responses to daily follow-up, treatment plans, and management procedures. Using IMS and EHR reduces error, increases communication between health practitioners, allows appropriate personnel to access the patient’s history, and increases the quality of care.

### 2.3. Expert Systems

An expert system allows human knowledge to be encoded into an automated system in a narrow domain [19]. To create an expert system, a set of assertions and a set of rules are collected that specify how an individual acts. Knowledge is interpreted in the form of rules known as either IF-THEN rules or production rules. In this study, COVID-19 management is integrated into the expert system using clinical guidelines (see Supplementary Materials S2). A patient is classified by the expert system automatically based on his/her medical condition, which is inserted into the EHR. Any knowledge-based expert system consists of several elements that are basic and simple: the knowledge base includes a set of facts, a set of rules, a knowledge engineer, and an inference engine. The life cycle of the development of the knowledge base consists of three phases: knowledge acquisition, knowledge verification and validation, and knowledge representation.

#### 2.3.1. Knowledge Acquisition

The acquisition of knowledge concerns the process of extracting, structuring, and organizing the domain knowledge from field experts into a development program [20]. Clinical guidelines remain valuable as a source of evidence-based medical knowledge. In this phase, the automating guidelines follow two processes: guideline selection and atomization [21]. First, the choice of which guidelines should be followed is an essential step in the development process, which can influence many of the following steps. The choice of which guidelines to apply—guidelines published by the Saudi MOH [22], WHO [23], the International Federation of Clinical Chemistry and Laboratory Medicine [24], and clinical practices [25]—is based on various factors: validity, evidence level, clinical applicability, institutional recommendations, variation in local practice, and the ease of operationalization (see the Supplementary Materials for COVID-19 diagnosis and management guidelines). In atomization, recommendations must be extracted and reduced to computable forms in the narrative text. The extraction of the guideline concepts was performed manually.

#### 2.3.2. Knowledge Verification and Validation

According to Peleg [26], the domain experts’ validation approach can be used when the domain knowledge discovers inconsistencies or mistakes during the validation and verification process. The specifications of the clinical guidelines are revised until they are validated and verified. They shall be safe for use. A loop can be observed in Figure 1. This phase focuses on quality assurance. Three domain experts perform the feedback process; the use of an odd number of experts allows any disagreements to be resolved by a voting majority. All comments are addressed after a review of the knowledge in order to confirm that it is correct. The knowledge will then be added into the validated knowledge base.

#### 2.3.3. Knowledge Representation

In hierarchical representations of data, the declarative representation of knowledge is used. The flowchart shows the knowledge derived from the clinical guidelines of COVID-19 diagnosis and management. Following an interpretation of the guidelines, a draw.io program was used to construct the rule diagram. In Supplementary Materials S2 Section 2.2, the management recommendations and the discontinuation of hospital isolation flowcharts published by the Saudi MOH are shown. The guidelines for the clinical classification and management of confirmed cases published by MOH [22] (Supplementary Materials S2 Section 2.1) were computerized in the form of clinical rules.

#### 2.3.4. Knowledge Engineer

An expert in AI language and knowledge representation—i.e., a knowledge engineer—examines a specific problem field, determines important concepts, and makes correct and effective representations of objects and relations in the field [20]. In Figure 1, the knowledge engineer’s role is demonstrated. He/she implements the life cycle of the development of knowledge based on the knowledge acquisition of a problem domain into knowledge representation.

#### 2.3.5. Inference Engine

The inference engine scheme consists of forward chaining. The fundamental idea of forward chaining is that, when data satisfies the premises of a rule (the if portion), the expert system asserts the conclusion of the rule (the then portion) as true [20]. The inferences are made on the website and the patient’s medical information is saved in the database as the EHR. The collected knowledge base is manipulated to prescribe the management plan.

### 2.4. Adaptive Learning

The expert system is integrated into the organization workflow and, in particular, with EHRs to adapt the new knowledge. The left side of Figure 1 displays the expert system with the existing knowledge, which is used before using the adaptation of the new knowledge and the incremental learning stage from zero. As shown on the right side, new knowledge can be obtained from two resources: new clinical guidelines or patients’ data from the EHR. The database contains information about patients admitted to hospital and their health history during hospitalization. Although the existing knowledge is used in the expert system, a knowledge discovery model can be created incrementally using patients’ records.

Adaptive learning includes two phases: knowledge verification and validation by human experts (Section 2.3.2) and revised knowledge (Section 2.4.2). Machine learning techniques are used for discovering knowledge from the EHR data (Section 2.4.1) and filtering the discovered knowledge to remove any old rules that existed in the validated knowledge base. The new knowledge after approval from human experts is transferred from the revised knowledge base to the validated knowledge base.

The clinical history, laboratory tests, and biochemical monitoring data are used by the machine learning technique (Section 2.4.1) to discover new knowledge, while the history of chest imaging is used to extract features and support the diagnosing process (Section 2.4.2). All the resulting knowledge will be revised by human experts (Section 2.4.2) before it is added to the validated knowledge base.

#### 2.4.1. Machine Learning

The patients’ data stored in the EHR can be analyzed by machine learning techniques. Patient data stored in the HER include clinical, biological, and laboratory result data at diagnosis and data regarding treatment that differs from the recommended treatment by WHO. After the database reaches 200 records, the data will be inserted into the classification and regression trees (CARTs) [27]. This approach is capable of explaining the relations between the laboratory findings of patients and treatment. The importance of a clear model to learn from the data stems from the need to trust the computation to extract new knowledge. In addition, clinicians must understand model recommendations to explain why the decision was made [28]. A CART is a repeating binary partition method that can process categorical and continuous data as indicators or outcomes. For the construction of the tree, the repetitive, splitting, and top-down-style conquering is used. Each node indicates a factor test, and each branch indicates a test result in which the leaf nodes represent classes. The data are partitioned recursively for selected factors. Here, the Gini Impurity Test is a splitting criterion used to determine the best split in the node. The classification of a patient with a choice tree takes place following a pathway through the tree of laboratory results to one of the leaves (treatment). This path from the tree root to a leaf prescribes conditions that every patient classified by the leaf must satisfy. Therefore, every leaf of a decision tree agrees with a clinical rule. The rules are in the following form:

IF factor A is category 1 AND factor B is category 2. THEN outcome 1.

The outcome is treatment, where factors A and B are the laboratory tests, category 1 and category 2 are the subcategories that belong to factors A and B, and the treatment is the class (lopinavir/ritonavir, ribavirin, interferon beta-1b, remdesivir, favipiravir, and tocilizumab). Nested cross-validation is used, so the training set is separated into training and validation, comprising 70%. The rest of the records are used for the test set. The model is trained on the training set, and performance is evaluated on the test set.

After the new clinical rule is formed, the rule appears on the knowledge inbox screen. The experts will be involved in the process of revising the knowledge (see Section 2.4.3).

#### 2.4.2. Radiomics Analysis for Chest Images

A chest x-ray (CXR) is used regularly to diagnose and monitor various respiratory disorders as a cost-efficient diagnostic test and is currently used as a method for the discrimination of COVID-19. Early stage progression and severe progression are the stages of the COVID-19 chest images (see the Supplementary Materials S1 Section 1.7, chest imaging). The radiomic features obtained from images taken by CXR can be used to detect and classify the COVID-19 stages. The heatmap of z-scores and the one-way ANOVA test [29] are used to find significant features that can be used to classify COVID-19 stages. A number of machine learning classifiers can be used for the best performance. The most accurate model can be used with the unseen data to classify COVID patients, and this model will be revised and validated by human experts to be included in the validated knowledge base.

#### 2.4.3. Revised Knowledge Base

This section includes the procedure of handling all the new knowledge in the form of clinical rules that are stored in the database (rule ID, rule explanation, number of usages, and weight). The clinical rules are filtered using the biased sampling (BiSAM) similarity measure [30]. The set of clinical rule pairs with similarities over some C thresholds are excluded. For example, if the rules occur in a validated knowledge base, the BiSAM algorithm generates weight W over C thresholds, while a low-W clinical rule is included in the knowledge inbox. This method requires that the filter stores a set of weighted samples in a database (rule ID, Paired rule ID, and weight), keeping track of the current W for each pair of rules seen.

The feedback is required by the human experts after the revised knowledge is updated with a set of new rules that come from the adaptive learner. The system will offer clinicians an explanatory summary of how patients have been managed during their hospitalization and how the system understands the test results. To validate the revised knowledge, the expert should give his/her agreement. The nature and imperfect knowledge of the medical decision-making process demands special techniques for dealing with fuzzy logic, which is defined by Zadeh [31]. The fuzzification process aims to determine the linguistic terms as fuzzy sets, while the degree of belief (DoB) as the membership function of each input belongs to one or more fuzzy sets (partial truth between 0 and 1). The linguistic terms of the feedback (low-, medium-, and high-strength recommendation) are based on the user selection, and the DoB depends on the number of uses of the rule. The weight of the knowledge represented by the fuzzy sets returns the DoB feedback. Once the DoB reaches a high degree and domain experts highly recommend the rule, it will move directly to the validated knowledge base.

### 2.5. Notification and Follow-Up System

In COVID-19, health practitioners have high workloads and other attention demands that disturb the prospective memory of a provider for follow-up tasks. In the system, health practitioners are notified about abnormal test results through EHR-based alerts. This concept is demonstrated in the Alert Watch and Response Engine (AWARE) software [32]. The system includes a range of priority alerts, such as abnormal chest imaging in response to abnormal hospital laboratory results. Notification also includes warning for symptoms that are examined twice daily for medical intervention.

### 2.6. Mobile Tracker

The system integrates Google Maps to locate the phone number of suspected cases so that places they have visited recently can be identified and notified of possible exposure to the virus. A JavaScript file contains all the mobile number data for suspected and confirmed cases after discharge and under isolation. For privacy concerns, this subproject can be conducted after getting the Saudi government’s approval to obtain mobile numbers recorded in the Tataman application and the HESN database to retrieve mobile location and service provider details.

### 2.7. Merging the Sub-Systems

In practice, the system can be developed as a website and merged with the sub-system development process in parallel. These sub-systems can be combined in a manner that allows the first to perform its activities in parallel with the second, and the second can provide feedback and important information to the first and vice-versa for activities. The website can be integrated and implemented using HTML, C#, ASP.net, and the SQL database in Microsoft Visual Studio 2019.

## 3. Results

### 3.1. Prototype of Interface Design

To demonstrate the prototype, a confirmed COVID-19 case is presented as an example: a 35-year-old man, who had a five-day history of cough, headache, and fever, after visiting his friend in Wuhan, China. Physical examination revealed a 39 °C body temperature, 134/87 mm Hg blood pressure, a pulse of 110 bpm, a breathing rate of 31 breaths per minute, and 96% oxygen saturation during the patient’s breathing. Later, the patient was administered nasopharyngeal and oropharyngeal swabs for 2019-nCoV by a real-time reverse transcriptase polymerase chain reaction (rRT-PCR) assay, and the result was positive. The patient reported a dry cough, two days of nausea and vomiting, diarrhea, and no shortness of breath. He had supportive care for symptom management. Treatment with 400/100 mg of Ritonavir every 12 h and 400 mg of ribavirin and interferon beta-1b every 12 h improved the clinical status of the patient. Laboratory testing was performed on the 3rd, 5th, 7th, 9th, and 10th days after admission, and a chest radiograph was taken on the 3rd, 5th, and 6th days in the hospital.

**Figure 2:**
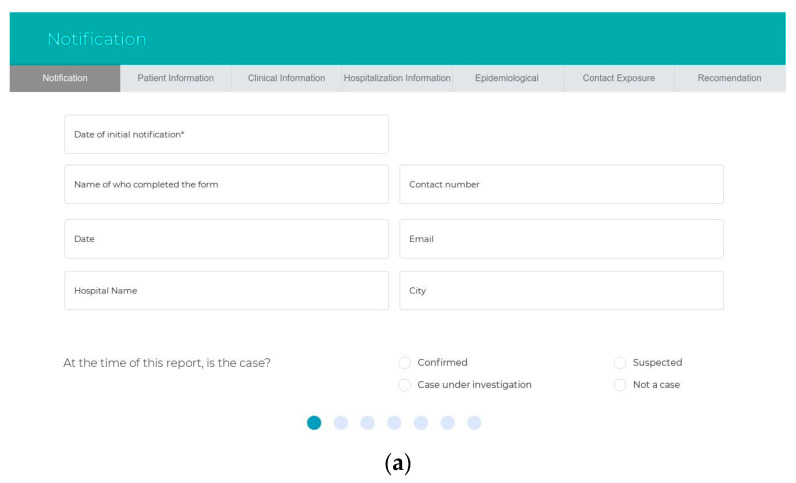

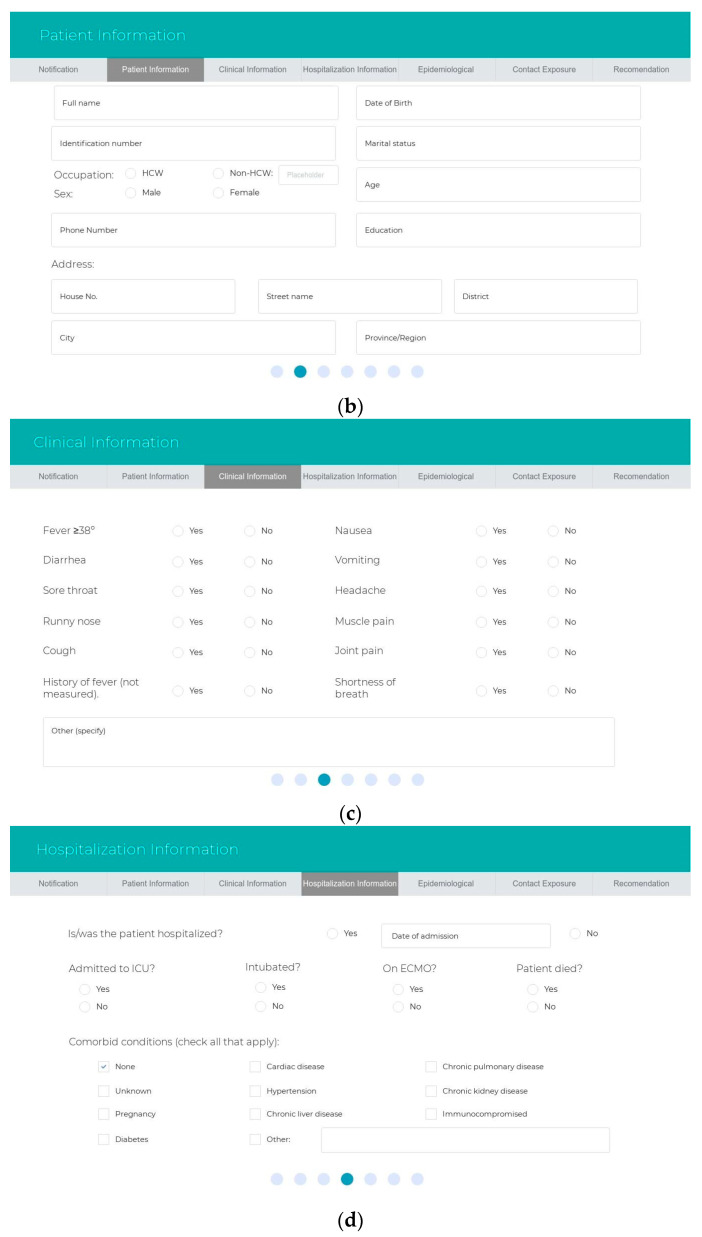

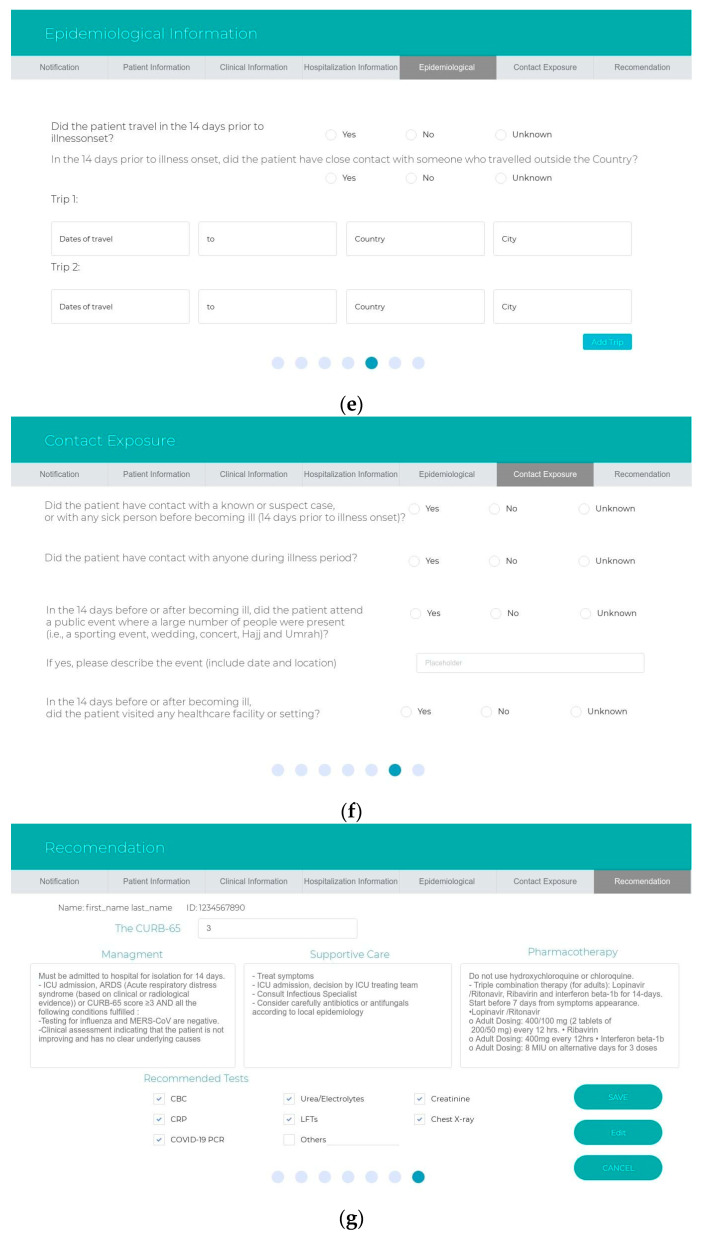
The prototype interfaces for the sub-system. The information management system: (**a**,**b**) patient information, (**c**) clinical data, (**d**) hospitalization, (**e**) epidemiological information, (**f**) contact exposure, and (**g**) expert system recommendation.

**Figure 3:**
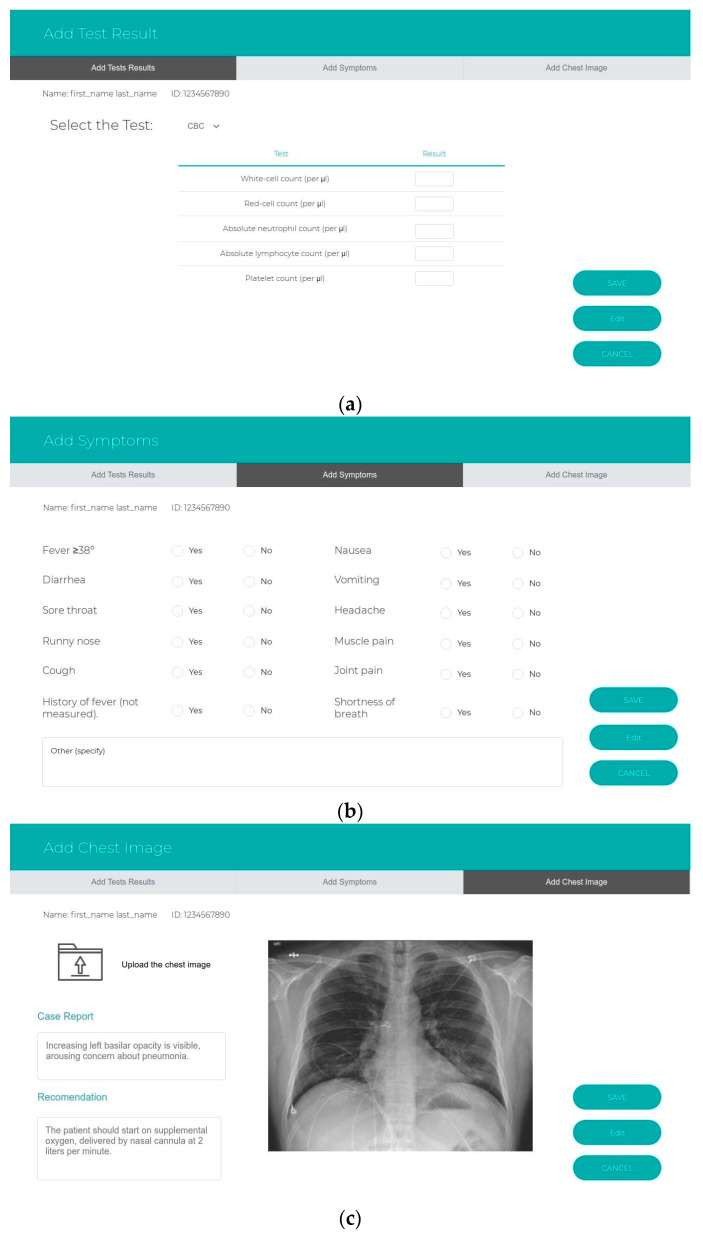
The prototype interfaces for the sub-system. The information management system: (**a**) add test results, (**b**) symptoms, (**c**) upload chest imaging.

**Figure 4:**
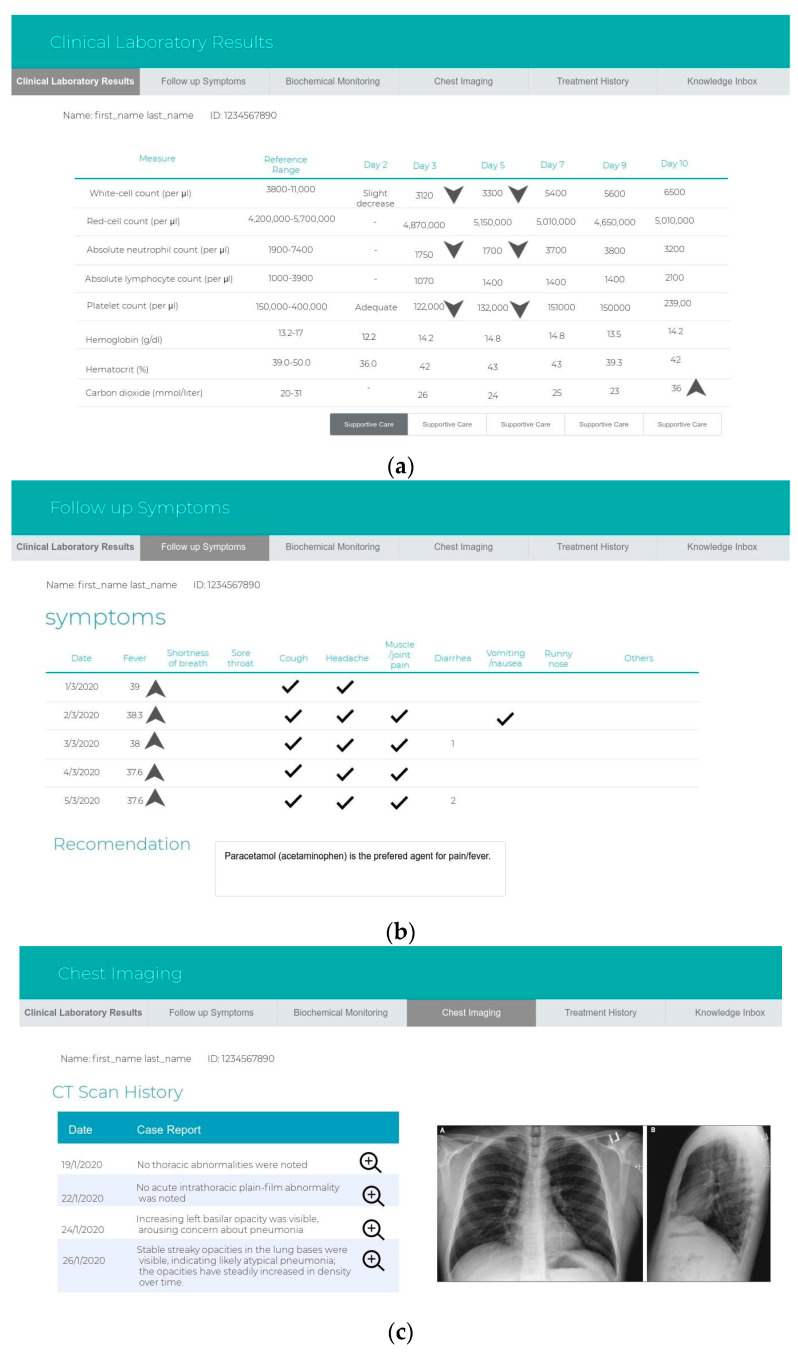

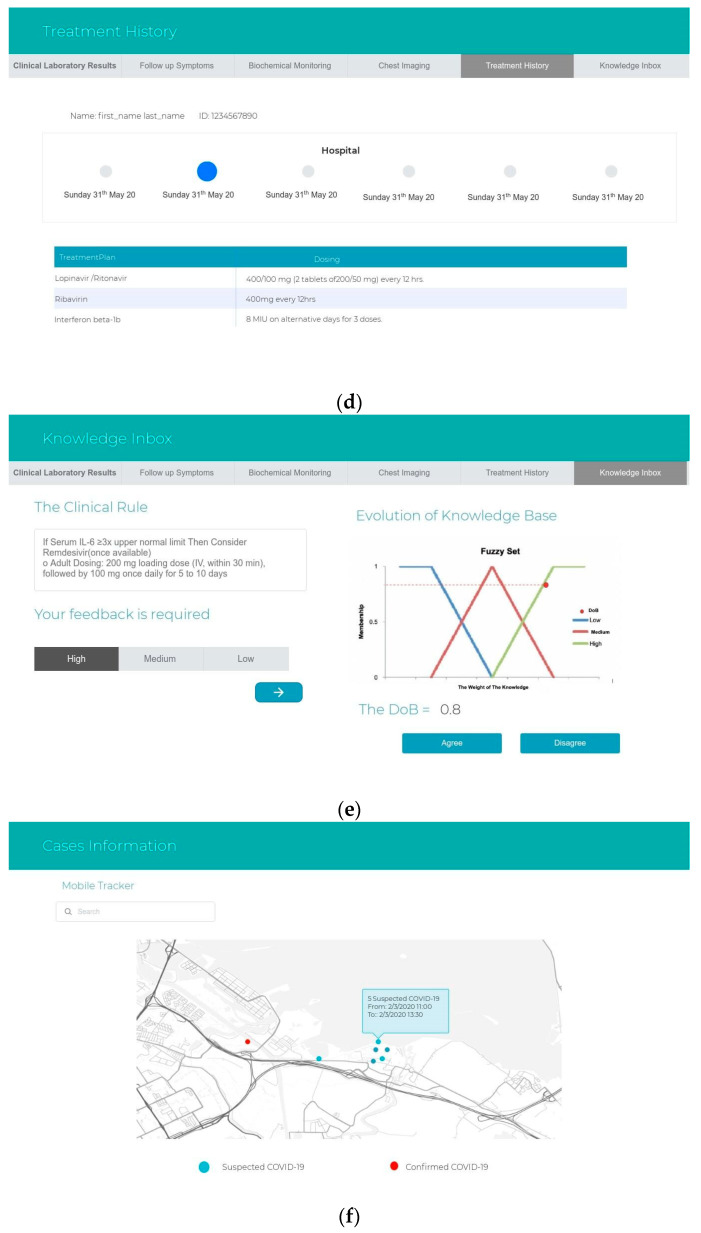
Prototype interfaces for the sub-system. The notification and follow-up system include (**a**) clinical laboratory results, (**b**) symptoms, (**c**) chest images, (**d**) treatment history, (**e**) the adaptive learning system and the new clinical rules in the knowledge inbox, and (**f**) the mobile tracker system.

The sub-systems prototype was designed based on the user types. The authorities for each user should be identified after logging into the website. There are five types of users in the system, and the hospital can decide who is responsible for using the system: health care workers, health care practitioners, patients, lab managers or radiologists, and healthy persons. First, health workers, such as nurses, can fill out a form that contains the patient information, the date of notification, the name of the person who completes the form, the hospital name, and whether the patient is a confirmed or suspected case or case under investigation. All the clinical information of the symptoms is required. Other details to assess the patient, such as hospitalization information, epidemiological information, contact exposure, and animal exposure, are also required. Health practitioners use an interface that displays the expert system recommendations in terms of the risk classification, the appropriate supportive care, recommended tests, and treatment recommendations for managing the case. The second interface displays the medical history, including all the tests, symptoms, and treatment from admission to discharge from the hospital, for viewing by the health care practitioner. In this interface, the alerts are displayed to catch the staff’s attention of any warnings. The third interface should be available when the health care practitioners add new treatments in managing the case. This interface shows the system evaluation of the new clinical rules and awaits the response from the experts (agree or disagree) before sending the new rules from the revised knowledge base into the validated knowledge base. Third, the patients use the interface remotely for daily follow-up of their symptoms. Fourth, health care personnel use the interface to insert any test results and chest images that are used. Finally, healthy individuals can use Google Maps to check on his/her destination—whether it is safe or it has been visited recently by suspected cases. Figure 2 and Figure 3 show the interfaces for the IMS and the expert system, while Figure 4 shows the prototype interfaces for the notification and follow-up, adaptive learning, and mobile tracker systems.

### 3.2. Task Automation

Several benefits could result from automating tasks. Firstly, reducing manual handling could reduce the errors after increasing health awareness education, support, and medical advice. Secondly, clinical guidance compliance can result, since the laboratory tests are recommended based on the WHO guidelines and are automatically requested by the expert system. Thirdly, the automated risk classification, triage checklist, recommended clinical action, and reported patients’ symptoms during the monitoring of health status reduce workload management. Finally, the proposed automation task could control the transmission of COVID-19 by exchanging paperwork and reducing the exchange cycle. Table 1 summarizes the tasks automated from the proposed system.

## 4. Discussion

This paper proposes a prototype for the diagnosis and management of COVID-19 cases. It can view the patient’s health information, enable health practitioners to access treatment recommendations and risk classification, and recommend test lists, as well as tracking test results and symptoms quickly. Upon its completion, such a system will help in decreasing health care professionals’ workload at the hospital; reducing the costs of health care; overcoming the shortage of trained health care professionals; and establishing universal health care coverage with accessibility guidelines, ensuring fair and efficient access that leads to improved diagnosis and management of COVID-19. By promoting scientific uniformity and consistency in health decisions and daily clinical practice, an intelligent system will allow for the management of various medical information items and the continuous update of medical records. According to Kwan et al. [33], the majority of clinical decision support approaches appear to produce small to moderate changes in particular treatment processes. They found in their systematic review that several factors could cause small to moderate changes. One of the factors is alert fatigue. However, the research shows that possible improvement mainly encompasses technical, medical, and economical areas. Technically speaking, the proposed system (i) computerizes the knowledge required from human experts and clinical guidelines, (ii) models and represents the knowledge in an appropriate manner for the implementation of the system, (iii) helps adaptive learners to make use of the integration of the new clinical guidelines with the EHR and health organizational workflow, and (iv) creates notifications and targeted content that trigger action at times of importance. Medically, the system (i) enhances the quality of care, (ii) limits differences in action, (iii) reduces the cost of medical care, (iv) supports clinicians in being effective and provides suitable recommendations when and where needed, (v) assists clinicians in deciding who to send to intensive care, (vi) provides prognoses according to the severity of risk, (vi) performs triages (home or hospital quarantine, hospitalization) for the patient at hospital admission, and (vii) follows up on patients’ daily symptoms remotely and suggests appropriate health care under the given circumstances. Economically, the system (i) overcomes the lack of trained health care professionals and the heavy reliance on foreign workers and (ii) makes sufficient skills and knowledge accessible to a large reserve of health practitioners. Thus, the proposed prototype has greater improvements in care, especially during the COVID-19 pandemic, where the burden of work increased without the use of the expert system as the number of confirmed cases increased. Therefore, the threat of alert fatigue is controlled in the proposed system because the Alert Watch and Response Engine (AWARE) software [32] includes a range of priority alerts. In addition, the proposed system recommends laboratory tests based on WHO guidance and reduces the workload of following up on results to determine outpatients’ outcomes. With the proposed design of the COVID-19 system, a health care professional needs only to check the highest priority cases and the related and recommended laboratory tests suggested by the expert system to manage a patient’s condition.

## 5. Conclusions

In the context of the coronavirus (COVID-19) global pandemic crisis, we present a diagnosis and management prototype using advanced intelligent technology to ensure the safety of the citizens and residents of the Kingdom of Saudi Arabia. The expert system paradigms that have been presented are for managing hospital patients and guiding clinicians and patients based on the medical knowledge approved by the Saudi MOH and the WHO. The previous field study showed that there is a shortage of specialists in the management of infections in Saudi Arabia because existing structures are not sufficient to control epidemics, both today and in the future. COVID-19 is a new disease, and the WHO is still updating the clinical guidelines as more scientific evidence and information become available. Therefore, a main part of our prototype that will satisfy the requirements of updating the knowledge base is the use of adaptive learning. Moreover, using an automated risk assessment for each case is essential to provide the right medical service for each patient.

The main advantages of the prototype consist of its ability to assist health practitioners with patients upon admission until after discharge from the hospital through controlling the spread of disease by managing the disease remotely and limiting direct contact with the patients. The availability of the computerized clinical guidelines and up-to-date knowledge base could also play an economic role; specifically, Saudi Arabia’s health organizations may not need to constantly train medical staff or to rely on foreign professionals because the expert system could provide all the information required for clinicians to handle the patients. In future work, we aim to proceed with the implementation of the proposed system. In addition, based on the same technology, in case of a need to handle any new epidemics, the expert system and its interface, as well as adaptive learning, can be used to build another system for other medical applications.

## Figures and Tables

**Table 1:** Tasks comparison between pre-automation and post-automation.

Task ID	Tasks	Pre-Automation	Post-Automation
1	Health awareness, education, support, and medical advice.	Hotline for health care workers for medical consultations.	Automatically inherited by the knowledge stored in the system.
2	Designated laboratory tests for confirmed cases.	To be selected after registering on the HESN website.	Recommended laboratory test by the expert system and based on the EHR tracking.
3	CURB-65 severity score.	Manual.	The score integrated in the expert system.
4	Visual Triage Checklist.	Risks scores are calculated manually for acute respiratory illnesses and reported using forms.	Integrated into the expert system.
5	Clinical action to manage the COVID-19 cases in mild, severe, and critical cases.	Manual.	The system automatically classifies patients in his/her risk category and recommends a suitable action and management plan.
6	List of the individuals who reported their symptoms during health status monitoring under quarantine.	Manually by health workers.	Up-to-date medical history displayed in timeline.
7	Healthy person to know the safe places to visit.	Not applicable.	Mobile tracker notifies a healthy person of any suspected cases in his/her destination.

## Supplementary Materials

The following are available online at https://www.mdpi.com/1660-4601/17/21/8066/s1: S1. Diagnosis; S2. COVID-19 management.

## References

[1] Yang X., Sun R., Zhao M., Chen E., Liu J. (2020). Expert recommendations on blood purification treatment protocol for patients with severe COVID-19. Recommendation and consensus. Chronic Dis. Transl. Med. Chin. Med. Assoc..

[2] WHO (2020). Coronavirus Disease (COVID-19) Dashboard. World Health Organization. https://covid19.who.int.

[3] Kondylakis H., Tsiknakis M. (2012). Computerized clinical guidelines: Current status & principles for future research. Studies Health Technol. Inform..

[4] Davenport T., Kalakota R. (2019). The potential for artificial intelligence in healthcare. Future Health J..

[5] Mujawar I., Swami S., Shikshan V. (2017). Comprehensive Study on Web Based Expert Systems for Disease. Int. J. Comput. Eng. Appl..

[6] Culebro J. (1993). Evolution of Expert System. Ph.D. Thesis.

[7] Nguyen T.T., Waurn G., Campus P. (2020). Artificial Intelligence in the Battle against Coronavirus (COVID-19): A Survey and Future Research Directions. Preprint.

[8] Medicine N., Medicine N., Imaging O., Iron W., Hospital S., Hospital N. (2020). Development of a Clinical Decision Support System for Severity Risk Prediction and Triage of COVID-19 Patients at Hospital Admission: An International Multicenter Study. Eur. Respir. J..

[9] Liu Y., Wang Z., Tian Y., Zhou M., Zhou T., Ye K. (2020). Design and development of COVID-19 risk assessment decision support system for general practitioners Table of Contents. J. Med. Internet Res..

[10] Salman F.M., Abu-Naser S.S. (2020). Expert System for COVID-19. Diagnosis.

[11] Saudi Arabia Government (2016). Saudi Vision 2030. Saudi National Transformation Program.

[12] Ministry of Health in Saudi Arabia (2017). Hesn. https://hesn.moh.gov.sa.

[13] Saudi Arabi Ministry of Health (2020). E-Services. https://www.moh.gov.sa/en/eServices/Pages/Rest-assured.aspx.

[14] Ministry of Health in Saudi Arabia (2020). Coronavirus Disease COVID-19 Guidelines.

[15] Tobaiqy M., Qashqary M., Al-Dahery S., Mujallad A., Hershan A., Kamal M., Helmi N. (2020). Therapeutic management of patients with COVID-19: A systematic review. Infect. Prev. Pr..

[16] (2020). Saudi MoH Protocol for Adults Patients Suspected of Confirmed with COVID-19. https://www.moh.gov.sa/Ministry/MediaCenter/Publications/Documents/MOH-therapeutic-protocol-for-COVID-19.pdf.

[17] Howell M.D., Donnino M.W., Talmor D., Clardy P., Ngo L., Shapiro N. (2007). Performance of Severity of Illness Scoring Systems in Emergency Department Patients with Infection. Acad. Emerg. Med..

[18] Deliège D.A., Debacker C., Smeesters S., Knani H., Neirynck I., De Clercq E. (2002). How to build an information system application to the health domain. Stud. Health Technol. Inform..

[19] Grosan Abraham C. (2011). Rule-based expert systems. Intelligent Systems.

[20] Shang Y. (2005). 5-Expert Systems.

[21] Tso G.J., Tu S.W., Oshiro C., Martins S., Ashcraft M., Yuen K.W., Wang D., Robinson A., Heidenreich P.A., Goldstein M.K. (2016). Automating Guidelines for Clinical Decision Support: Knowledge Engineering and Implementation. AMIA Annu. Symp. Proc..

[22] Ministry of Health in Saudi Arabia (2020). Coronavirus Disease COVID-19 Guidelines. https://www.moh.gov.sa/en/HealthAwareness/EducationalContent/PublicHealth/Pages/corona.aspx.

[23] Livingston E., Bucher K., Rekito A. (2020). Coronavirus Disease 2019 and Influenza 2019–2020. JAMA.

[24] Varming K., Forsum U., Bruunshuus I., Olesen H. (2004). International Federation of Clinical Chemistry and Laboratory Medicine. EJIFCC.

[25] Emergency Medicine Cases (2020). A Practical COVID-19 Resource for Emergency Medicine. https://emergencymedicinecases.com/covid-19-updates/.

[26] Peleg M. (2013). Computer-interpretable clinical guidelines: A methodological review. J. Biomed. Inform..

[27] Van Ryzin J., Breiman L., Friedman J.H., Olshen R.A., Stone C.J. (1986). Classification and Regression Trees. J. Am. Stat. Assoc..

[28] Freitas A.A. (2013). Comprehensible classification models: A position paper. ACM SIGKDD Explor..

[29] Tamal M., Alshammari M., Alabdullah M., Hourani R., Abu Alola H., Hegazi T.M. (2020). An Integrated Framework with Machine Learning and Radiomics for Accurate and Rapid Early Diagnosis of COVID-19 from Chest X-ray. medRxiv.

[30] Campagna A., Pagh R. (2011). Finding associations and computing similarity via biased pair sampling. Knowl. Inf. Syst..

[31] Zadeh L. (1994). Soft computing and fuzzy logic. IEEE Softw..

[32] Smith M.W., Murphy D., Laxmisan A., Sittig D., Reis B., Esquivel A., Singh H. (2013). Developing software to “track and catch” missed follow-up of abnormal test results in a complex sociotechnical environment. Appl. Clin. Inform..

[33] Kwan J.L., Lo L., Ferguson J., Goldberg H., Diaz-Martinez J.P., Tomlinson G., Grimshaw J.M., Shojania K.G. (2020). Computerised clinical decision support systems and absolute improvements in care: Meta-analysis of controlled clinical trials. BMJ.

