# Supplementary material for: Prototype Development of an Expert System of Computerized Clinical Guidelines for COVID-19 Diagnosis and Management in Saudi Arabia
Source: Int J Environ Res Public Health. 2020 Nov 2;17(21):8066. doi: 10.3390/ijerph17218066 (PMC7662618; doi:10.3390/ijerph17218066)
Supplement: Supplementary file 1 [file ijerph-17-08066-s001.pdf]

International Journal of
Environmental Research and Public Health

MDPI

Prototype Development of an Expert System to Computerized Clinical Guideline for COVID-19 Diagnosis and Management in Saudi Arabia

Haneen Reda Banjar [1,*], Heba Alkhatabi [2,3], Nofe Alganmi [1,3] and Ghaidaa Ibraheem Almouhana [1]

1 Computer Science Department, Faculty of Computing and Information Technology, King Abdulaziz University, Jeddah 80200, Saudi Arabia; nalghanimi@kau.edu.sa (N.A.); galmouhana@stu.kau.edu.sa (G.I.A.)
2 Department of Medical Laboratory Technology, Faculty of Applied Medical Science, King Abdulaziz University, Jeddah 80200, Saudi Arabia; halkhattabi@kau.edu.sa
3 Center of Excellence in Genomic Medicine Research (CEGMR), King Abdulaziz University, Jeddah 80200, Saudi Arabia
* Correspondence: hrbanjar@kau.edu.sa

Received: 29 September 2020; Accepted: 29 October 2020; Published: date

S1. Diagnosis

1.1Suspected cases

As figure 1 shows the management recommendation by the ministry of health in Saudi Arabia:

- All suspected cases must be tested initially with Covid-19 PCR.
- If suspected case is clinically stable, home isolation or isolation in designated facility may be considered based on assessment by public health team and CCC until result become available.
- If patient is clinically unstable, must be admitted to hospital for isolation for 14 days.
- If result of initial test is positive, patient is considered as confirmed case.

1.2 Confirmed patients

1.2.1 Symptomatic

- Re- testing must be done patient is clinically recovered
- If result is positive, then repeat the test every 72 hours.
- In order to discontinue hospital isolation precautions as shown in figure 2 , two negative samples 24 hours apart are required for all patients. After discharge, patients are recommended to continue 14 days of home isolation.

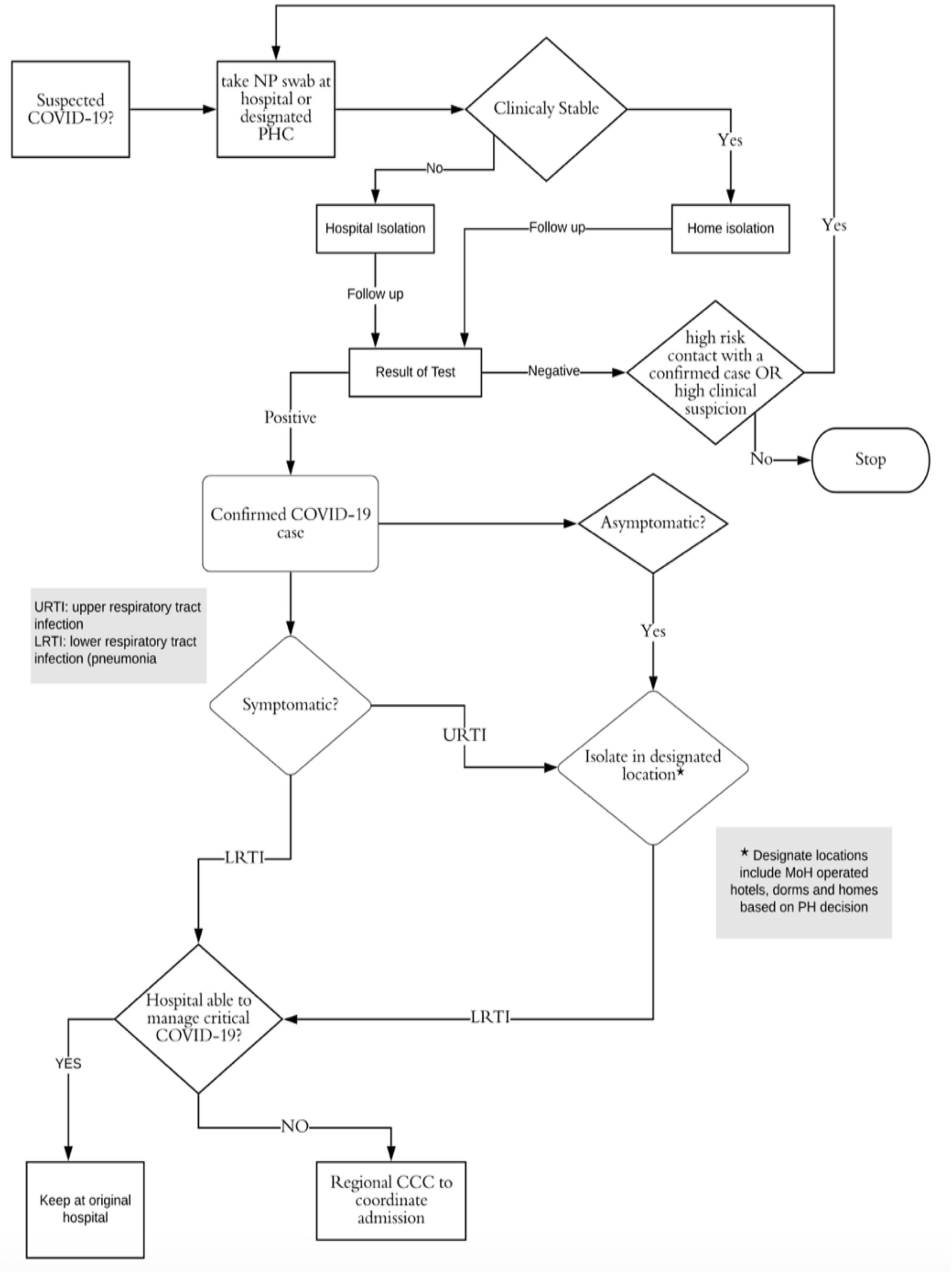

-

Figure 1. *Management recommendation by the Saudi MOH[2]*

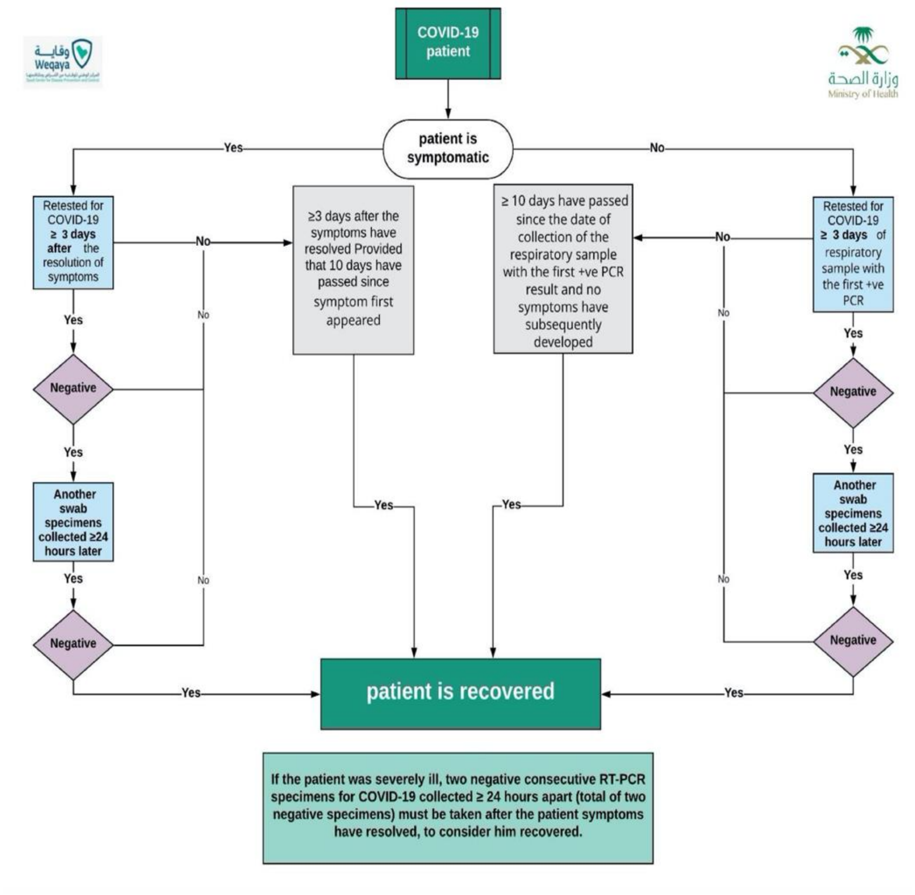

Figure 2. *Discontinuation of hospital isolation[2]*

1.2.2 Asymptomatic

- Re- testing must be done every 72 hours after confirmation
- In order to discontinue designated facility isolation precautions, two negative respiratory samples 24 hours apart are required for all patients. After discharge, patients are recommended to continue 14 days of home isolation.

1.3 Epidemiological History

- Had a history of travel abroad
- Travel to an identified high-risk area in the kingdom
- A close physical contact prior to symptom onset with a confirmed ("Close Contact´ is defined as:

1. Health care associated exposure, including providing direct care for COVID-19 patients, working with HCWs infected with COVID-19, visiting patients or staying in the same close environment of a COVID-19 patient.
2. Working together in close proximity or sharing the same classroom environment a with COVID-19 patient.
3. Traveling together with COVID-19 patient in any kind of transportation.
4. Living in the same household as a COVID-19 patient.

- COVID-19 case* (A confirmed case is defined as a suspected case with laboratory confirmation of COVID-19 infection).
- Working in or attended a healthcare facility where patients with confirmed COVID-19 are admitted.

1.4 Symptoms

- Fever
- Cough
- Chills
- Fatigue
- Nausea
- Vomiting
- Diarrhea
- Sore throat
- Headache
- Runny nose
- Muscle pain
- Joint pain
- Shortness of breath
- Abnormalities in smell and/or taste
- Some patients may have aches and pains,
- Nasal congestion
- malaise
- dyspnoea
- Symptoms for sever cases:
- Dyspnea and/or hypoxemia after one week
- Older and/or immunosuppressed patients:
- Atypical symptoms (e.g., falls, delirium, confusion, functional decline).
- Acute myocardial infarction
- Venous thromboembolism

1.5 Laboratory Tests

The following table 1 shows the parameters collected from laboratory tests and its clinical and biological interpretation[1].

Table 1. the parameters collected from laboratory tests

Parameter	Result	Clinical and biological interpretation
**Neutrophil count***	Increased	Bacterial (super)infection “Neutrophilia”
**WBC***	Decreased	
**Lymphocyte count***	Decreased	Decreased immunological response to the virus “Lymphonpenia”
**Erythrocyte sedimentation rate**	Increased	
**leukocyte**	Increased	Bacterial (super)infection “Leukocytosis”
**MDW (monocyte volume distribution width)**	Increased	Severe viral infection/viremia/viral sepsis
**Platelet***	Decrease	Consumption (disseminated) coagulopathy “Thrombocytopenia”
**C-reactive protein***	Increased	Severe viral infection/viremia/viral sepsis
**Procalcitonin***	Increased	Bacterial (super)infection

**Albumin***	Decreased	Impairment of liver function
**Liver enzymes (GOT (AST), GPT (ALT), GGT, ALP, Bilirubin)**ǀ*	Increased	Liver injury
**Lactate dehydrogenase (LDH)***	Increased	Pulmonary injury and/or widespread organ damage
**Kidney parameters (Creatinine, Urea/BUN)***	Increased	Kidney injury
**Lactate**	Increased	
**CK-MB**	Increased	Cardiac injury
**Myoglobin**	Increased	Cardiac injury
**Troponin***	Increased	Cardiac injury
**D-dimer***	Increased	Activation of blood coagulation and/or disseminated coagulopathy
**Prothrombin time (sec)***	Increased	Activation of blood coagulation and/or disseminated coagulopathy
**Ferritine***	Increased	Sever inflammation
**Cytokines (IL-6)***	Increased	Cytokine storm syndrome
**Blood gases(ABG)***	Estimated Modification	Important in clinical care management
**Pulse oximetry may reveal oxygen saturation (SpO_2)**	Low <90%	

For procalcitonin and coagulation tests a special mention shall be made. The previous test does not appear to be altered significantly in COVID-19 patients upon admission, but the incremental increase in value tends to represent a worse pronouncement. Table 2 and table 3 shows the critical / sever adult and children cases consideration.

Table 2 . Critical / sever adult cases consideration

Parameter	Abnormalities
**Lymphocytes**	decrease progressively
**IL-6 and C-reactive proteins,**	Increase progressively
**Lactate**	Increases progressively
**Lung lesions**	Develop rapidly in a short period of time

Table 3. Critical / sever children cases consideration

Parameter	Abnormalities
**Respiratory rate**	Increase
**Poor mental reaction and drowsiness**	-
**Lactate**	increases progressively
**Infiltration on both sides or multiple lobes, pleural effusion and Lung lesions**	develop rapidly in a short period of time
**under the age of 3 months who have either underlying diseases (congenital heart disease, bronchopulmonary dysplasia, respiratory tract deformity, abnormal hemoglobin, and severe malnutrition, etc.) or immune deficiency or hypofunction (long-term use of immunosuppressants).**	Considered sever

1.6 Biochemical Monitoring

In vitro diagnostic testing, biochemical monitoring of COVID-19 patients is essential for the assessment of disease severity and progression, and for the monitoring of treatment interventions. A recommended test list based on current literature is included above in the table 1 and have been marked with * along with the major laboratory abnormalities associated with adult COVID-19 patients and their clinical indications potential[1]. In addition to more common laboratory tests, new evidence suggests that cytokine storm syndrome may be at risk in patients with severe COVID-19. Where appropriate to diagnose severe patients suspected of hyperinflammation, cytokine tests, in particular IL-6 should be used. The most common abnormalities include higher C-reactive protein (CRP) levels, erythrocyte sedimentation rate (ESR), lactate dehydrogenase (LDH) and D-dimer.For pediatric patients, as opposed to adults, the laboratory profile is not explicit and tends to be in line with SARS in severe COVID-19 pediatric patients.Interpretation of laboratory tests for children based on known adult trends is not recommended, as pediatric data and features are lacking[1].

1.7 Chest Imaging

Chest imagery of pregnant women is considered safe.
The following table 4 shows the stages of COVID-19 and the chest image findings.

Table 4. Chest image findings and the stages of the COVID-19

Stage	Image finding
**Early stage**	Multiple small patchy shadows and interstitial changes, apparent in the outer lateral zone of lungs.
**Progression**	Multiple ground glass opacities and infiltration in both lungs.
**Sever**	Pulmonary consolidation may occur while pleural effusion is rare.<br>chest imaging that showed obvious lesion progression within 24-48 hours >50%.

1.8 Recovery Signs

The neutrophilia resolved, and the other blood indicators were back to normal ranges.

1.9 Risk classification

The following information in table 5 is used to classify the confirmed cases at hospital[2].
The CURB-65 score parameters are: Confusion, Urea nitrogen, Respiratory rate, Blood pressure, and 65 years of age and older.
For each of the following features present, the CURB-65 severity score is 1 point:

Confusion (mental test score 8 new disorientation in person, place or time)
BUN > 20 mg/dL
Respiratory rate 30 breaths/min
Blood pressure (systolic <90 mm Hg, or diastolic 60 mm Hg)
Age 65 years.

Table 5. CURB-65 classification

Score	Risk	Disposition
0 or 1	1.5% mortality	Outpatient care
2	9.2% mortality	Inpatient vs. observation admission
≥ 3	22% mortality	Inpatient admission with consideration for ICU admission with score of 4 or 5

S2. COVID-19 Management

2.1 Clinical Classification and Management of Confirmed Cases

The following table 6 shows the classification based on the guidelines from WHO and ministry of health is Saudi Arabia[2], [3]. Figure 3, Figure 4 and Figure 5 show the rule diagrams for COVID-19 management after classification and their precautions and pharmacotherapy. There are two cases: suspicious and confirmed. Finally, Figure 6 and Figure 7 show the management of thromboprophylaxis in adults and pediatric patients.

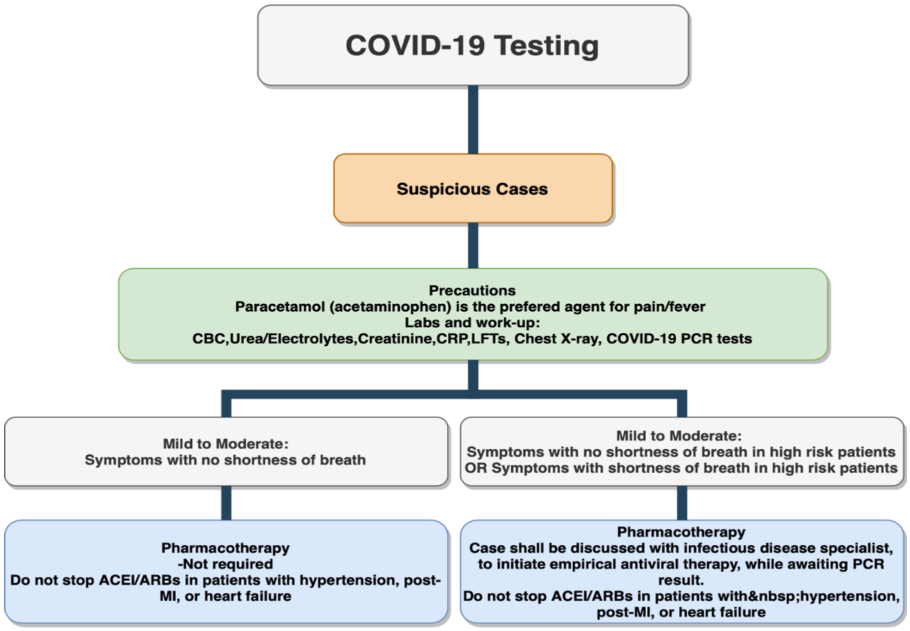

Figure 3. Suspected cases management

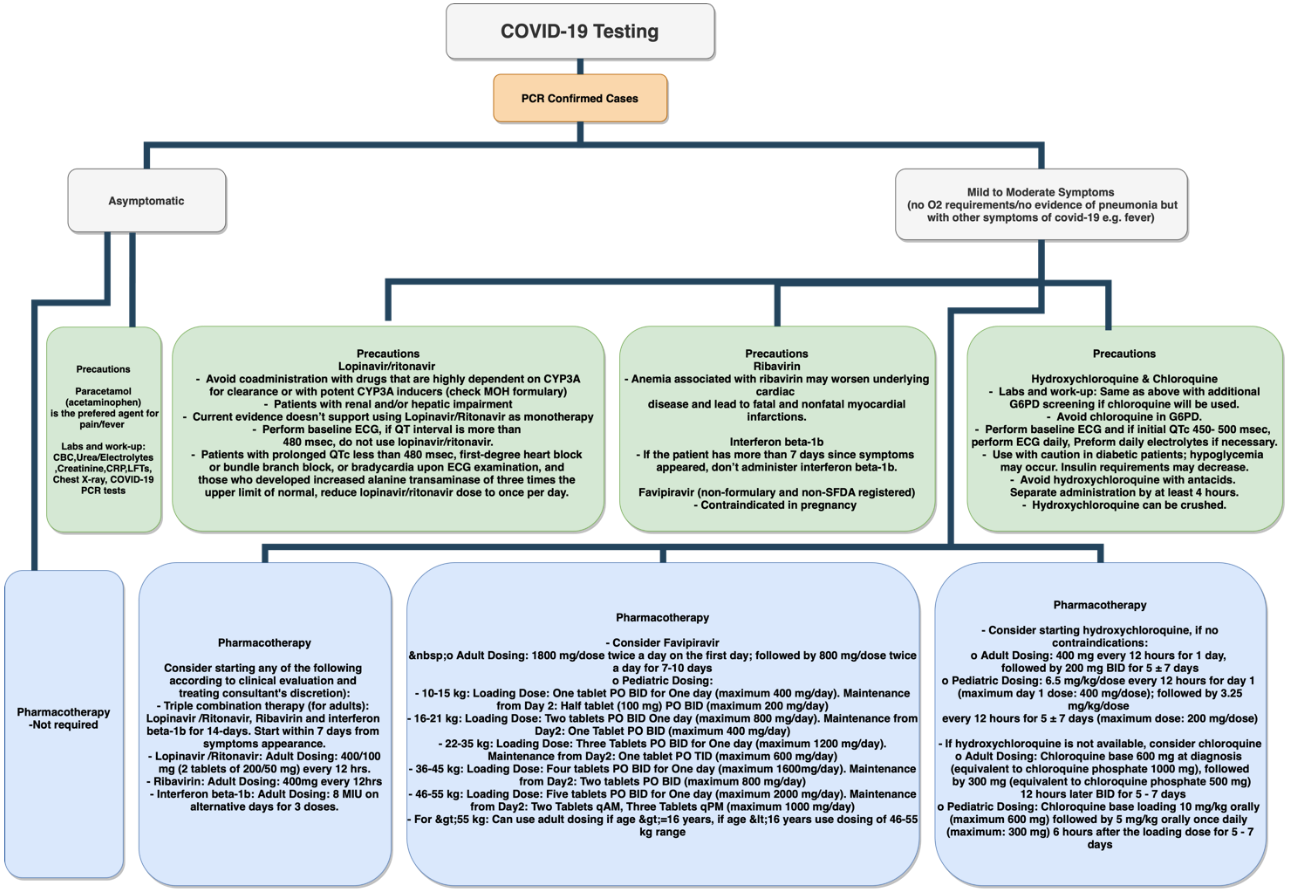

Figure 4. Confirmed cases management in asymptomatic and mild to moderate symptoms

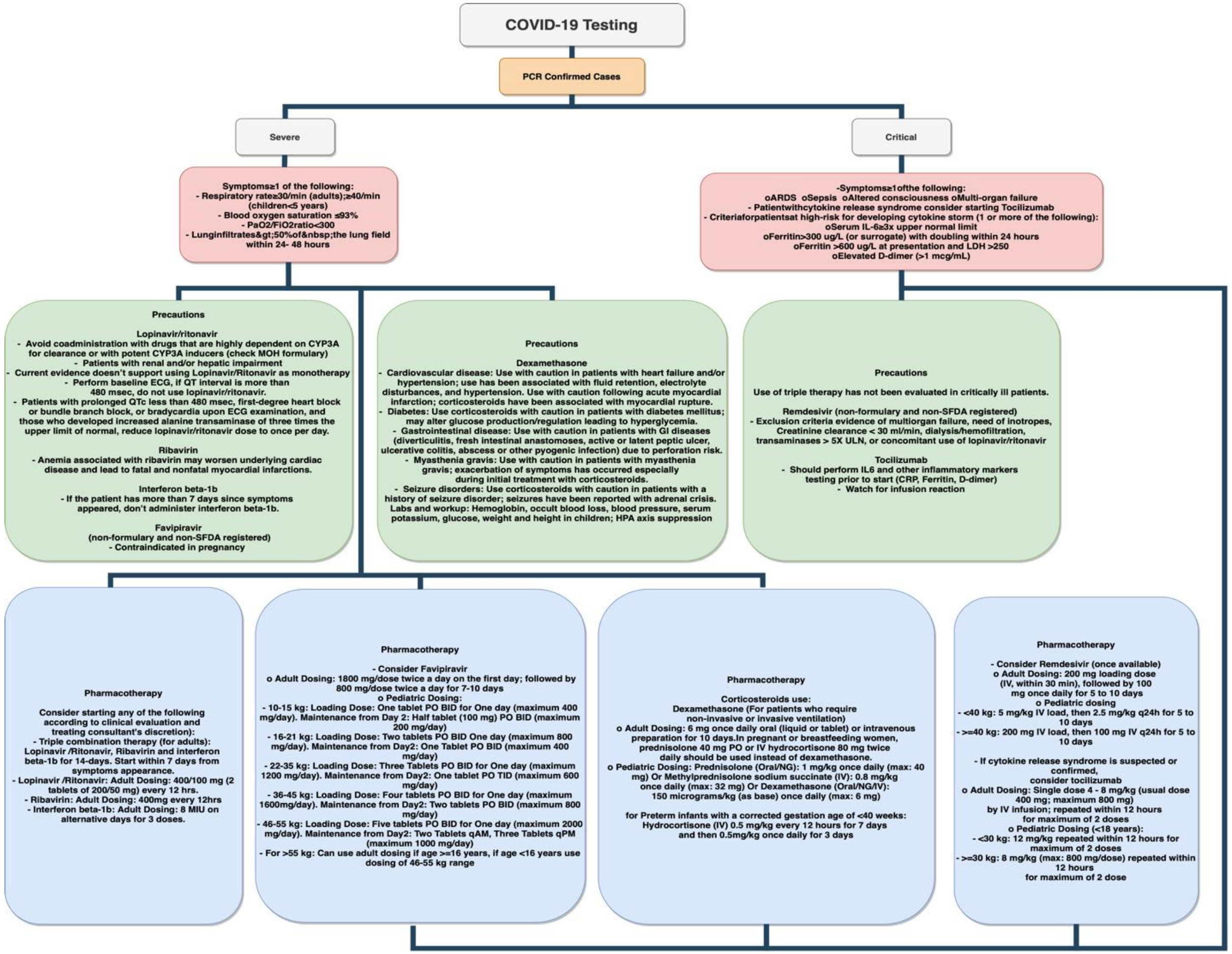

Figure 5. Management in severe and critical cases

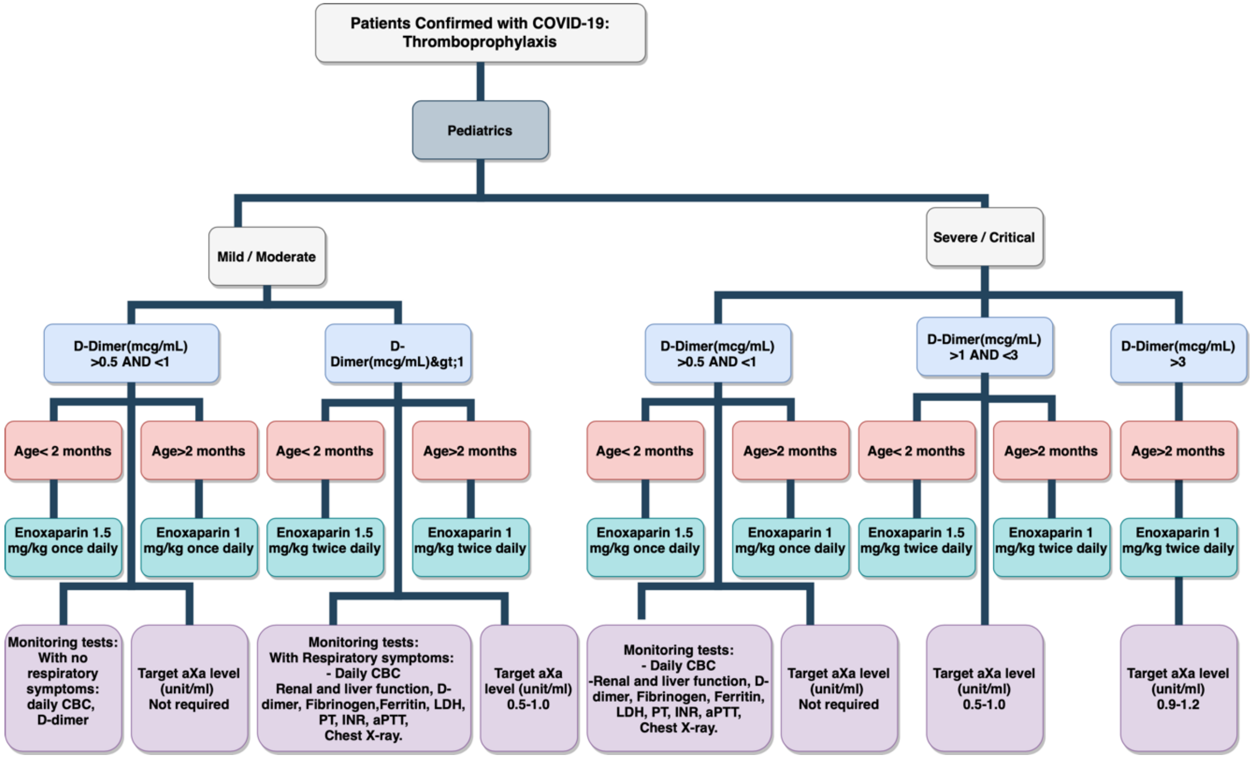

Figure 6. Thromboprophylaxis management in pediatric cases

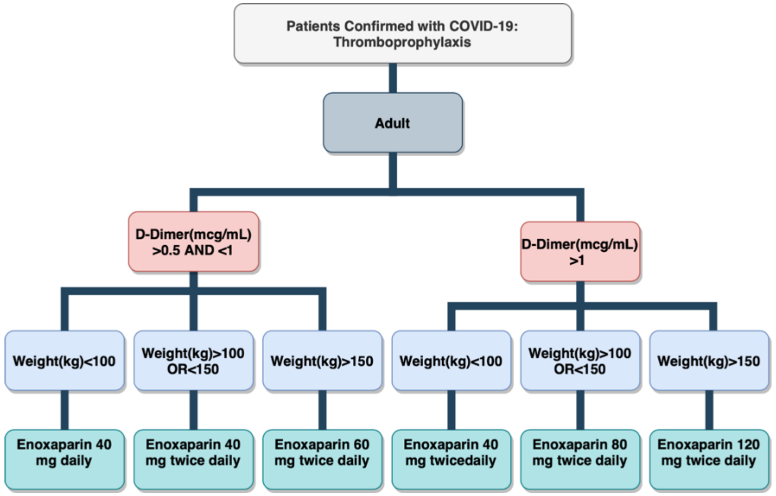

Figure 7. Thromboprophylaxis management in adult case

Table 6. COVID-19 management, source[2], [4], [5]

Diagnosis	Symptoms	Management	Supportive care
**Mild**	-If the patient have mild symptoms and no risk factors for poor outcome (age >60 years, cases with underlying co-morbidities, e.g., chronic cardiovascular disease, chronic respiratory disease, diabetes, cancer).<br>- Mild cases with risk factors for poor outcomes outcome (age >60 years, cases with underlying comorbidities, e.g., chronic cardiovascular disease, chronic respiratory disease, diabetes, cancer)	Home isolation or isolation in designated facilities may be considered based on assessment by public health team and CCC.<br>-Must be admitted to hospital for isolation for 14 days	-Treat symptoms.<br>-Consult Infectious Disease Specialist
**Moderate cases**	Showing fever and respiratory symptoms with radiological findings of pneumonia		
**Sever cases (Adult)**	metabolic acidosis that is difficult to correct, arrhythmias, acute cardiac injury, shock, coagulopathy,<br>Patient with ≥ 1 of the following symptoms:<br>(1) Respiratory distress (≧30 breaths/ min)<br>(2) Oxygen saturation≤93% at rest;<br>(3) Arterial partial pressure of oxygen (PaO2)/ fraction of inspired oxygen (FiO2)≦ 300mmHg (l mmHg=0.133kPa).<br>Chest imaging that showed obvious lesion progression within 24-48 hours >50% (Lung infiltration >50% of the lung field within 24-48 hours)	Must be admitted to hospital for isolation for 14 days.<br>- ICU admission, ARDS (Acute respiratory distress syndrome (based on clinical or radiological evidence)) or CURB-65 score ≥3 AND all the following conditions fulfilled :<br>-Testing for influenza and MERS-CoV are negative.<br>-Clinical assessment indicating that the patient is not improving and has no clear underlying causes	- Treat symptoms<br>- ICU admission, decision by ICU treating team<br>- Consult Infectious Specialist<br>- Consider carefully antibiotics or antifungals according to local epidemiology

Diagnosis	Symptoms	Management	Supportive care
**Critical cases**	-ICU admission, requiring mechanical ventilation;	Patients admitted to ICU if the prognosis for recovery and quality of life is acceptable, taken into account factors such	-Patient should be discharged from the ICU to a lower acuity area when a patient's physiologic status has

	-Patient requiring more than 2 hours on Non-Invasive Ventilation (NIV) or High Flow Nasal Cannula (HFNC).<br>-Respiratory Distress<br>Need O2 > 6 LPM to maintain SpO2 > 92 or PaO2 > 65.<br>Rapid escalation of oxygen requirement.<br>Significant work of breathing i.e. Tachypnea.<br>-Patient with hemodynamic instability despite initial conservative fluid resuscitation<br>-Patient require vasopressor support.<br>-Patient with a decreased level of consciousness.<br>-Acidosis<br>ABG with pH < 7.3 or PCO2 > 50 or above patient's baseline.<br>Lactate > 2.<br>-Patient with more than one organ failure.<br>-Patient requires continuous renal preplacement therapy CRRT and cannot tolerate hemodialysis.<br>-Patient with unstable vital signs not yet on vasopressors.	as age, comorbidities, prognosis, underlying diagnosis, and treatment modalities that can influence survival.<br><br>If the ICU bed is unavailable, ICU physicians continue to deliver care for critical care patients in the emergency department or the ward with the help of the primary team.	stabilized, and they are no longer a need for ICU monitoring and treatment.<br>-Discharge parameters are based on ICU admission criteria, the admitting criteria for the next lower level of care, institutional availability of these resources, patient prognosis, physiologic stability, and ongoing active interventions.<br>-Stable vital signs and other hemodynamic parameters without intravenous inotropic/ vasopressor support.<br>-Patients on low dose inotropic support (less than 5 mcg/kg/minute of Dopamine) may be discharged earlier if ICU bed is required during the crisis.<br>Normal or baseline level of consciousness.<br>-Stable respiratory status, normal airway patency, and normal work of breathing.<br>-At least 24 hours post-extubation if the patient was on mechanical ventilation.<br>-No or controlled cardiac dysrhythmias.<br>-No frequent suctioning requirement for a patient on chronic mechanical ventilation.<br>-No frequent suctioning requirement for a patient with tracheostomy.<br>-Patients with no escalation decision and (DNR).
**Elder**	Older people are more likely to have extensive lung lobe involvement, interstitial changes, and pleural thickening compared with younger patients.	Older patients and those with comorbidities, such as cardiovascular disease and diabetes mellitus, have increased risk of severe disease and mortality. They may present with mild symptoms but have high risk	Early detection of inappropriate medication prescriptions is recommended to prevent adverse drug events and drug interactions for those being treated for COVID-19.

		of deterioration and should be admitted to a designated unit for close monitoring.<br><br>Physiological changes with age lead to declines in intrinsic capacity, manifested as malnutrition, cognitive decline, and depressive symptoms; those conditions should be managed comprehensively.	Older people are at greater risk of polypharmacy, as a result of newly prescribed medications, inadequate medication reconciliation, and a lack of coordination of care, all of which increases the risk of negative health consequences.

**Diagnosis**	**Symptoms**	**Management**	**Supportive care**
**Children and neonate**	Clinical findings in neonate, especially in premature infants, are non-specific Therefore, it is important to closely monitor vital signs<br>-Respiratory and cardiovascular signs may include tachypnea, grunting, nasal flaring, increased work of breathing (WOB), apnea, cough, or tachycardia.<br>-Other findings may include poor feeding, lethargy, vomiting, loose stools, and abdominal distension.<br><br>Children in general:<br>fever, cough, sore throat, nasal congestion, sneezing, and rhinorrhoe, gastrointestinal symptoms such as vomiting and diarrhea particularly newborns and infants , or only manifested as low spirits and shortness of breath.<br>Relatively mild.<br>(1) Tachypnea (RR ≥ 60 breaths/min for infants aged below 2 months; RR ≥ 50 BPM for infants aged 2-12 months; RR ≥ 40 BPM	-Healthy asymptomatic neonate born at or near term who does not require neonatal intensive care.<br>-Symptomatic or high-risk neonates requiring neonatal intensive care.<br>-The neonate should be transferred into the designated transport incubator without undergoing any non-urgent neonatal care.<br>-All non-urgent neonatal care and examination should be carried out in the isolation room e.g. weighing, immunization.<br>-All neonates should be separated from their mothers with NO SKIN TO SKIN contact.<br>-The equipment used should undergo terminal cleaning or disposed of based on universal recommendations following a biohazard decontamination protocol.<br>-If a single room is not available, or if the COVID exposed infant census requires cohorting, infants should be maintained at least 6 feet apart and/or placed in air temperature-controlled incubator.	If the mother's result is tested negative for COVID-19 infection:<br>1. Breast feeding and rooming in with mother is allowed.<br>2. Discharge the baby with follow up.<br>3. The neonate must be monitored until Day 28 of life.<br>If the mother's result is tested positive for COVID – 19 infection: 1. Collect the Oro /nasopharyngeal swab for the newborn at birth. 2. Continue with routine care.<br>3. If the neonate result is tested negative born to a mother with confirmed COVID 19 infection (asymptomatic and stable), discharge once negative for two consecutive samples to a COVID – 19 negative caregivers.<br>If the neonate result is tested positive born to a mother with confirmed COVID 19 infection (asymptomatic and stable):<br>1.Continue close monitoring and routine care management.<br>2. Repeat the sample every 48 - 72 hours until the result turns negative 3. Discharge

	for children aged 1-5 years, and RR ≥ 30 BPM for children above 5 years old)<br>) independent of fever and crying;<br>(2) Oxygen saturation ≤ 92% on finger pulse oximeter taken at rest;<br>(3) Labored breathing (moaning, nasal fluttering, and infrasternal, supraclavicular and intercostal retraction), cyanosis, and intermittent apnea;<br>(4) Lethargy and convulsion;<br>(5) Difficulty feeding and signs of dehydration.	-Symptomatic Neonate requiring Neonatal Intensive Care: Medical management is according to neonatal unit guidelines in consultation with pediatric infectious disease team<br><br>-Laboratory and Radiological tests:<br>1. CBC, CRP, Blood Culture<br>2. Blood gas analysis, acid-base studies<br>3. Serum electrolytes, liver and kidney function with cardiac biomarkers<br>4. Chest radiograph. Lung ultrasound is recommended<br>5. Other investigations as needed	once negative for two consecutive samples to a COVID – 19 negative caregivers.<br>4. Plan for frequent follow-up through 14 days after birth including parent's education.

Diagnosis	Symptoms	Management	Supportive care
**Pregnant**	The clinical course of pregnant women is similar to that of patients of the same age.	-Delivery should occur in an isolated room and the room should be disinfected right after the patient is discharged to the ward following the infection control measures.<br>-Continuous fetal monitoring during labour.	Pregnancy<br>-Oral paracetamol is considered safe in normal therapeutic doses for short-term use as a minor analgesic/antipyretic in pregnancy<br>-Consider Administering IV paracetamol to a pregnant woman only if clearly needed.<br>Lactation:

	-Normal vaginal delivery with delaying rupture of membranes is advised.<br>-Caesarean section is for obstetrical reasons. E. Labor, delivery and recovery should be done in the same room.<br>-New born babies of covid-19 infected mother should not be allowed to be in contact with their mother's till the mother is cured or declared free of the disease.<br>-Pregnant patients with suspected covid-19 infection will be dealt with as if they are infected.	Oral is considered safe, For IV paracetamol, consider the need based on the clinical diagnosis or condition<br><br>Pregnancy:<br>Fetal risk cannot be ruled out. Fetal ocular toxicity have been reported.<br>Lactation:<br>-No adverse effects of Hydroxychloroquine in infants exposed during the lactation period have been observed. Although the benefits of breastfeeding outweigh the theoretical risk to the infant, the nursing infant should always be monitored for adverse effects.<br><br>Pregnancy:<br>-Fetal risk cannot be ruled out. Fetal ocular toxicity have been reported<br>-Administer chloroquine during pregnancy only if the potential maternal benefit outweighs the potential fetal risk<br>Lactation:<br>-WHO consider chloroquine compatible during breastfeeding.<br>-WHO recommends against use in G6PD-deficient infants, and advises monitoring premature infants and neonates for side effects such as hemolysis and jaundice.<br>-Due to the potential for adverse events in the nursing infant, advise the nursing mother to either discontinue nursing or discontinue chloroquine therapy, considering the clinical benefit of the drug to the mother.

2.4 Chest Imaging

The decision is based on the following figure 8 which shows the chest imaging test results can assist in the COVID-19 management.

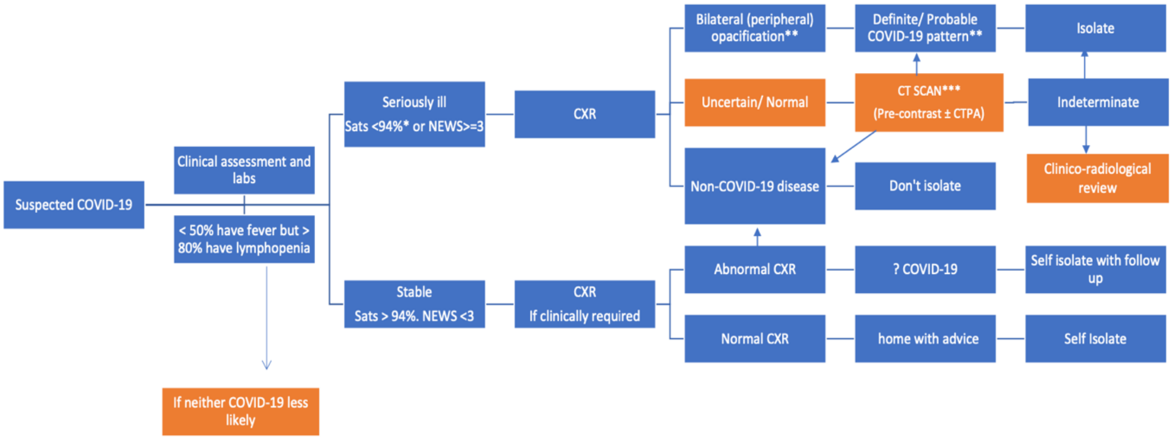

Figure 8. Radiology decision tool for suspected COVID-19.* 94% unless known COPD in which case <90%. ** Unsuspected/ unexpected cases may be incidentally discovered on CXR/ CT at this stage; should be reviewed in the context of clinical suspicion as to likelihood of COVID-19. ***Classic and Indeterminate CTs should be scored either: 'mild' or 'moderate/severe' source is[3].

2.5 Intensive Care Unit (ICU) Management

Canadian expert opinion demonstrated in ICU management for Reuben Strayer's oxygenation strategy algorithm [6]. ICU management for Rueben Strayer's oxygenation strategy algorithm. Examples of protocols, checklists and algorithms are intended for education, requiring modification and approval by Saudi hospital before being used in clinical practice.

2.6 Mechanical Ventilation Protocol for COVID-19

In the European society of intensive care medicine and the guide for prone position of awake patient with COVID 19 [6], these guidelines were based on the latest European Society of Intensive Care Medicine (ESICM), survival sepsis Campaign panel guidelines, as well as expert opinions that maintain the best practices taking account of local resources, cultural changes, previous local practices, and expertise.

References

[1] "IFCC Information Guide on COVID-19," *International Federation of Clinical Chemistry and Laboratory Medicine*, 2020. .

[2] "Coronavirus Disease COVID-19 Guidelines," *Ministry of Health in Saudi Arabia*, 2020. [Online]. Available: https://www.moh.gov.sa/en/HealthAwareness/EducationalContent/PublicHealth/Pages/corona.aspx. [Accessed: 20-May-2020].

[3] "Radiology decision tool for suspected COVID-19," *British Society of Thoracic Imaging*, 2020. .

[4] "Coronavirus disease 2019 (COVID-19)," *World Heal. Organ.*, vol. 2019, no. March, p. 2633, 2020, doi: 10.1001/jama.2020.2633.

[5] I. Ali and O. M. L. Alharbi, "COVID-19: Disease, management, treatment, and social impact," *Sci. Total Environ.*, vol. 728, p. 138861, 2020, doi: https://doi.org/10.1016/j.scitotenv.2020.138861.

[6] "A practical COVID-19 resource for Emergency Medicine," *Emergency Medicine Cases*, 2020. [Online]. Available: https://emergencymedicinecases.com/covid-19-updates/. [Accessed: 21-May-2020].

Publisher's Note: MDPI stays neutral with regard to jurisdictional claims in published maps and institutional affiliations.

© 2020 by the authors. Submitted for possible open access publication under the terms and conditions of the Creative Commons Attribution (CC BY) license (http://creativecommons.org/licenses/by/4.0/).
